# Cryotherapy and pain intensity during endodontic treatment of mandibular first permanent molars with symptomatic irreversible pulpitis: A randomized controlled trial

**DOI:** 10.1007/s00784-023-05084-1

**Published:** 2023-06-03

**Authors:** Ahmad Abdel Hamid Elheeny, Dania Ibrahem Sermani, Esteer Azer Saliab, Mohammed Turky

**Affiliations:** 1 Paediatric and Community Dentistry, Faculty of Dentistry, Minia University, Minya, Egypt; 2 Minia University Dental Hospital, Faculty of Dentistry Hospital of Minia University, Minya, Egypt; 3 Faculty of Dentistry, Minia University, Minya, Egypt

**Keywords:** Cryotherapy, Pain, Symptomatic irreversible pulpitis

## Abstract

**Objectives:**

The study aimed to assess the effectiveness of cryotherapy application after inferior alveolar nerve block (IANB) administration of the mandibular first permanent molars with symptomatic irreversible pulpitis (SIP) in adolescence. The secondary outcome was to compare the need for supplemental intraligamentary injection (ILI).

**Materials and methods:**

The study was designed as a randomized clinical trial including 152 participants aged from 10 to 17 years who were randomly assigned to two equal groups; cryotherapy plus IANB (intervention group) and the control group (conventional INAB). Both groups received 3.6 mL of 4% articaine. For the intervention group, ice packs were applied in the buccal vestibule of the mandibular first permanent molar for 5 min. Endodontic procedures started after 20 min for efficiently anesthetized teeth. The intraoperative pain intensity was measured using the visual analogue scale (VAS). The Mann–Whitney (*U*) and chi-square tests were applied to analyze data. The significance level was set to 0.05.

**Results:**

There was a significant reduction in the overall intraoperative VAS mean in the cryotherapy group compared to that in the control group (*p* = 0.004). The success rate was significantly higher in the cryotherapy group (59.2%) compared to the control group (40.8%). The frequency of extra ILI was 50% and 67.1% in the cryotherapy and control groups, respectively (*p* = 0.032).

**Conclusions:**

The cryotherapy application boosted the efficacy of pulpal anesthesia of the mandibular first permanent molars with SIP in patients below the age of 18 years. Additional anesthesia was still necessary for optimal control over pain.

**Clinical relevance:**

Pain control during endodontic treatment of primary molars with irreversible pulpitis (IP) is a significant factor in a child’s behavior in the dental office. Although the inferior alveolar nerve block (IANB) is the most commonly used technique to anaesthetize mandibular dentition, we found its success rate to be relatively low during endodontic treatment of primary molars with IP. Cryotherapy is a new approach that significantly improves the efficacy of IANB.

**Clinical trial registration:**

The trial was registered at ClinicalTrials.gov (reference no. NCT05267847).

**Supplementary Information:**

The online version contains supplementary material available at 10.1007/s00784-023-05084-1.

## Introduction

The inferior alveolar nerve block (IANB) is the routine and standard technique used to obtain pulpal anesthesia of the mandibular quadrant [1]. The mandibular first permanent molar is the most common tooth that requires endodontic intervention [2]. Several factors play a crucial role in the success rate of the IANB. Anatomical variations related to the nerve include the nerve course, the mandibular foramen location, and accessory nerve supply [3]. Other factors are related to the patients, including various pain thresholds, preoperative pain intensity, and the preexisting propagation of pulp tissue inflammation [3, 4].

According to reports, the IANB success rate ranged from 10 to 75% [5, 6]. Other reports addressed a range of 14 to 57% [7, 8]. This indicates a high failure rate for IANB of mandibular teeth with SIP. To enhance the success rate of IANB in mandibular teeth with SIP, several approaches have been considered. For instance, the administration of supplementary local anesthesia, testing different anesthetic drugs, increasing the volume of anesthetic solution or the vasoconstrictor concentration [9], the use of various mandibular block techniques [10], and the administration of preoperative systemic analgesics [11].

Cold application is a well-known approach for postoperative pain management in medicine [10]. The physiologic effect of cryotherapy application is derived from its ability to diminish the local inflammatory response, slow down the nerve impulse, spread, and minimizes hemorrhage and postoperative edema [12]. In endodontics, postoperative pain was assessed after root canal irrigation with cold saline following mechanical instrumentation [13, 14]. To the best of the author's knowledge, the use of the cold application after mandibular blocks was tested exclusively in one previous study conducted by Topçuoglu et al. [10]. They reported a significant enhancement of pulpal anesthesia in terms of no or mild pain after the cryotherapy application, with a success rate of 55.8% compared to the standard INAB’s 30.8%.

Very limited data was available regarding the effectiveness of IANB after cryotherapy application in patients with SIP. The available data was limited to patients over 18 years. Therefore, the primary aim of this trial was to assess the effectiveness of cryotherapy application after IANB injection of the mandibular first permanent molars with SIP in adolescents (i.e., the null hypothesis (*H0*) of the primary and secondary outcomes suggested no difference in the success rate of IANB after cryotherapy application and the standard nerve block technique). The secondary goal was to compare the need for supplementary intraligamentary injection (ILI) in the cryotherapy and control groups.

## Materials and methods

### Ethical approval

Clinical procedures were launched after obtaining written informed consent from each participant’s legal guardian. All procedures in studies involving human participants were performed in accordance with the ethical standards of the institutional and/or national research committee and with the 1964 Helsinki Declaration and its later amendments or comparable ethical standards. The trial was approved by the Research Ethics Committee of the Faculty of Dentistry in Minia University (reference number 602).

### Design and sample size

The study was designed as a randomized controlled trial with two parallel arms. A pilot study included 36 participants who were equally distributed into two groups to estimate the proportional difference. At an alpha level of significance of 5% and a power of 80%, the sample size was calculated based on the following formula: *N* $=$ 2 * (*Z*α + *Z*β) ^2^ $\times$ *p*_1_ (1 – *p*_1_) + *p*_2_ (1 – p_2_)/∆^2^. The success rates of the cryotherapy plus IANB group (*p*_1_) and conventional IANB group (*p*_2_) were 0.67 and 0.44, respectively. The required sample size was 152 patients (76 per group) after adding 10% to compensate for potential attrition.

### Randomization, allocation, and blinding

The randomization process was implemented using the following electronic website: https://www.sealedenvelope.com/. To obtain two balanced groups with an allocation ratio of 1:1, an independent investigator implemented block randomization with a block size of 4. Based on the treatment type, 152 opaque, adequately sealed, identical envelopes contained standardized printed papers. The envelopes were encoded for either the intervention or control group (76 per group) and placed in two separate, identical plastic containers. At the time of the procedures, an independent nurse selected two envelopes from each container. The four selected envelopes were shuffled [15]. The participant’s legal guardian selected one envelope randomly. Only the chief investigator was aware of the interpretation of the treatment codes [16]. Endodontic treatment was performed for the participants after receiving either IANB plus cryotherapy application (intervention group) or standard IANB. Three independent experts in endodontic treatment and pediatric dentistry with a minimum of 14 years of experience implemented the IANB injection, cryotherapy application, and endodontic treatment [10].

### Eligibility criteria

#### Inclusion criteria

##### Patient-related criteria

- Healthy 10- to 17-year-old children who were classified as classes I or II according to the American Society of Anesthesiologists were included.
- Body weight of a minimum of 35 kg.

##### Tooth-related criteria

Presence of a restorable mandibular first permanent molar with signs and symptoms of SIP and a history of spontaneous lingered pain precipitated by thermal stimuli [17]. To confirm the diagnosis of SIP, an electric pulp test (EPT; Parkell D640 Digitest II Pulp Vitality Tester) and thermal cold stimuli were used. The tooth isolation was performed using cotton rolls, and then dried with sterile gauze. For EPT, the probe tip was coated with toothpaste. For cold pulp testing, a cotton pellet was soaked with Green Endo-Ice refrigerant (Coltene/Whalkedent Inc. OH, USA) and applied for 5 s. The buccal surface’s middle third was the chosen site for the EPT probe and cold cotton application. To provide a base for comparison, both the electrical and thermal stimuli were applied on the contralateral mandibular first permanent molar. Irreversible pulpitis was confirmed in the case of an exaggerated response to a cold stimulus and an early response to the EPT at a low current flow).

- The SIP diagnosis was confirmed, via the early response to the electric pulp test (EPT) and the positive response to thermal pulp testing [18].
- A periapical radiograph revealed a closed apical foramen in a mandibular first permanent molar (class 5 according to Cvek’s stages of root development).
- Negative findings of preoperative periapical radiographs with intact periodontal ligament space.
- Only patients who have arrived at the clinic and rated their pain intensity as moderate or severe according to the visual analogue scale (VAS) preoperatively (i.e., before IANB injection) [10].

#### Exclusion criteria

##### Patient-related criteria

- Severe emotional, behavioral, or intellectual disorders

##### Tooth-related criteria

- Gingival swelling, fistulous or sinus tract, abnormal tooth mobility, and pain on biting or percussion are all clinical signs of pulp necrosis or apical periodontitis.
- History of chronic pain.
- Radiographic evidence of periapical radiolucency, internal or external root resorption, and/or pulp stones or calcification.
- Previous endodontic treatment (e.g., pulpotomy or pulpectomy) [19].
- Preoperative analgesic intake within the last 12 h [10].

### Clinical procedures

#### Control group (conventional IANB)

Topical anesthesia 20% benzocaine (I-Gel Topical Anesthetic gel) was applied for one minute. The IANB site of injection was three-quarters of an imaginary line extending back from the coronoid notch midpoint to the deepest portion of the pterygomandibular raphe [1]. The syringe barrel was placed in the mouth corner on the contralateral side, above the primary molars or premolars [1]. After approximately two-thirds of the penetration depth of a 27-gauge long needle (31 mm) contacted the bone, negative aspiration was confirmed. Two cartridges (3.6 mL) of articaine hydrochloride 4%, and epinephrine, 1:100,000 (Septocaine®, SEPTODONT Ltd) were deposited slowly (1 mL/min) [20]. Lingual soft tissues were anesthetized to allow placement of the rubber dam clamp with a few drops deposited during needle retraction.

#### Intervention group (IANB plus cryotherapy)

Silicon ice blocks were filled with water and placed in the freezer. The temperature was monitored using a digital freezer/fridge thermometer (Gellvann, China). The ice packs were used when their temperature ranged between − 4 and 0 °C to avoid any risk of frostbite. If the ice pack was removed before 5 min of continuous application, the patient was excluded.

After the IANB injection, ice packs wrapped in sterile gauze were applied to the buccal vestibule of the mandibular first permanent molar with SIP for 5 min. Participants were asked to remove the ice packs immediately once an intolerant coldness or burning sensation had been felt [10].

#### Checking the effectiveness of anesthesia

The effectiveness of LA was checked 20 min following IANB administration. Both subjective and objective indicators were identified. Numbness of the lower lip and tongue, indicating that the inferior alveolar nerve and tongue have been anesthetized [1].

Objective testing using the EPT (Parkell D640 Digitest II Pulp Vitality Tester) at two successive intervals (2 min apart) and a thermal cold stimulus with a cotton pellet damped with Green Endo-Ice refrigerant; Coltene/Whalkedent Inc. OH, USA) for 5 s was confirmed [10]. Only efficiently anesthetized teeth (i.e., negative responses to the maximum output of EPT and to thermal cold stimuli), the cavity was accessed. Otherwise, the patient was precluded from the study.

#### Endodontic treatment procedures

All endodontic procedures were conducted in one visit (i.e., single-visit endodontic treatment). After caries excavation, a straight-line access cavity was gained with a sterile round carbide bur and Endo-Z bur. Coronal pulp tissues were removed and root canal orifices were located. To determine the working length, a suitable-sized hand K-file (MANI Inc. Tochigi, Japan) was used to engage the whole root canal length up to the apical constriction. A periapical radiograph image was used to estimate the working length (1 mm safety allowance shorter than the radiographic apex) [19]. To confirm the working length, an electronic apex locator (Root ZX mini, MORITA was used. Adopting the step-back technique, 3 successive manual K-files completed the mechanical instrumentation with a minimum of ISO tip No. 30. The irrigation protocol was implemented for each root canal in the following order: (*i*) 10 mL 3% sodium hypochlorite (NaOCl) (Hyposol, Prevest DenPro) with 30-gauge side-vented needles (Endo-Top®; PPH CERKAMED) adjusted to the working length minus 1 mm was used during mechanical preparation (5 mL) and for final irrigation after finishing the root canal instrumentation (5 mL), (*ii*) 2 mL 17% EDTA solution (Prevest DenPro) to remove the smear layer, and (*iii*) final irrigation with 5 mL 0.9% saline [21]. After finishing the chemomechanical instrumentation, root canals were dried and filled. Root canals were filled with gutta-percha (Diadent Group International) using the lateral condensation technique and sealed with a resin-based root canal sealer (ADSEAL, Meta Biomed Co.). A postoperative periapical radiograph was taken to check the quality of the root canal filling. The access cavity was sealed with a glass ionomer layer (Equia TM Fil, GC) of 2–3 mm thickness. Finally, composite resin (Tetric N Ceram Bulk Fill, Ivoclar, Vikvadent AG) filled the cavity.

### Pain intensity assessment tool

Pain intensity was assessed using the visual analogue scale (VAS), which is represented by a 100 mm (10 cm) horizontal line with one end referring to no pain (score 0) and the other referring to the worst pain (score 10). The patient was asked to choose a mark that reflected his/her feelings of pain severity. Pain intensity was classified into 4 levels: no pain (0), mild pain (1–3), moderate pain (4–6), and severe pain (7–10) [21–26]. Pain assessment was performed on two occasions; first, before starting the treatment (participants who rated no or mild pain were excluded). Second, during access to the cavity, patients were asked to report any painful sensation, and treatment was halted instantly. Participants who experienced any discomfort (mild, moderate, or severe pain) during endodontic treatment received a 0.5-mL supplementary ILI.

### Statistical data analysis

Data were analyzed using IBM SPSS, Version 20 Inc., Chicago, Ill software. Data were tested for normality using the Kolmogorov–Smimov and Shapiro–Wilk tests. For normally distributed continuous data, Independent sample *t* test. The non-parametric preoperative and intraoperative VAS scores were tested using Mann–Whitney (*U*). A chi-square test was used to analyze categorical data (gender, need for supplementary anesthesia, which was classified as “yes” or “no”, and success rate (no or mild pain) [10]. The alpha level of significance was set to 0.05.

## Results

**Fig. 1:**
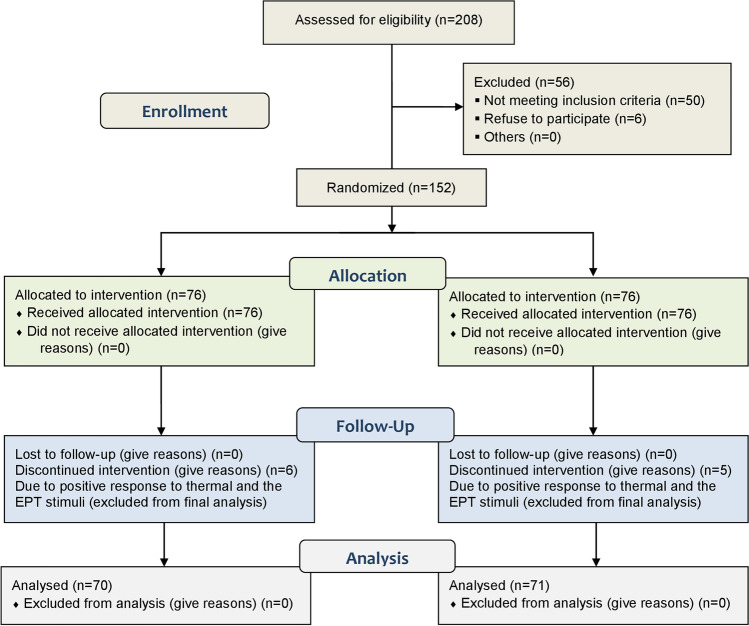
CONSORT flow chart of the trial

Of the 208 subjects assessed for eligibility, 152 participants were enrolled after excluding 56 subjects (50 did not fulfill the inclusion requirements, and 6 refused to participate). Elven subjects were excluded (6 in the intervention group and 5 in the control group) because their teeth responded positively to the EPT and thermal tests (Fig. 1). There was no statistically significant difference between the participants' demographic data (i.e., gender and age) and preoperative VAS means. A significant reduction in the overall intraoperative VAS scores in the cryotherapy group compared to that in the control group (*p* = 0.004). For participants who recorded preoperative moderate pain intensities, their intraoperative VAS scores were significantly lower in the cryotherapy group (*p* = 0.002). While, no significant difference between the intervention and control groups was found in the case of severe preoperative VAS scores (*p* = 0.142). The success rate (no pain/mild pain) was significantly higher in the cryotherapy group (59.2%) compared to the control group (40.8%) (*p* = 0.023) (Table 1).

**Table 1:** Participants’ baselines, preoperative and intraoperative VAS mean and median, and success rate

Predictors	IANB + cryotherapy *N* (%)	Control group *N* (%)	*P* value
Gender			
Male	36(47.4)	31(40.8)	0.41^†^
Female	39(51.3)	45(59.2)	
Age (years)			
Mean (SD)	13.93(2.21)	14.09(2.10)	0.67^‡^
Preoperative VAS			
Moderate pain			
Mean (SD)	5.00(0.89)	5.02(0.91)	0.97^§^
Median (IQR)	5(2)	5(2)	
Severe pain			
Mean (SD)	8.28(0.90)	8.54(0.65)	0.18^§^
Median (IQR)	8(1)	8(1)	
Overall preoperative pain			
Mean (SD)	6.08(1.79)	6.22(1.87)	0.69^§^
Median (IQR)	6(4)	6(3)	
Intraoperative VAS			
Moderate pain			
Mean (SD)	1.82(2.14)	3.50(3.02)	0.002^§^
Median (IQR)	0(4)	5(6)	
Severe pain			
Mean (SD)	4.56(2.65)	5.65(2.80)	0.14^§^
Median (IQR)	5(4)	6(5.25)	
Overall intraoperative pain			
Mean (SD)	2.84(2.61)	4.24(3.10)	0.004^§^
Median (IQR)	2(5)	5(5)	
Success rate	45(59.2)	31(40.8)	0.02^†^

^†^ Chi-square test; ^‡^Student *t* test; ^§^ Mann–Whitney *U* test; *P* value was set to 0.05

*VAS* Visual analogue scale, *IANB* inferior alveolar nerve block, *SD* standard deviation; *IQR* interquartile range

**Fig. 2:**
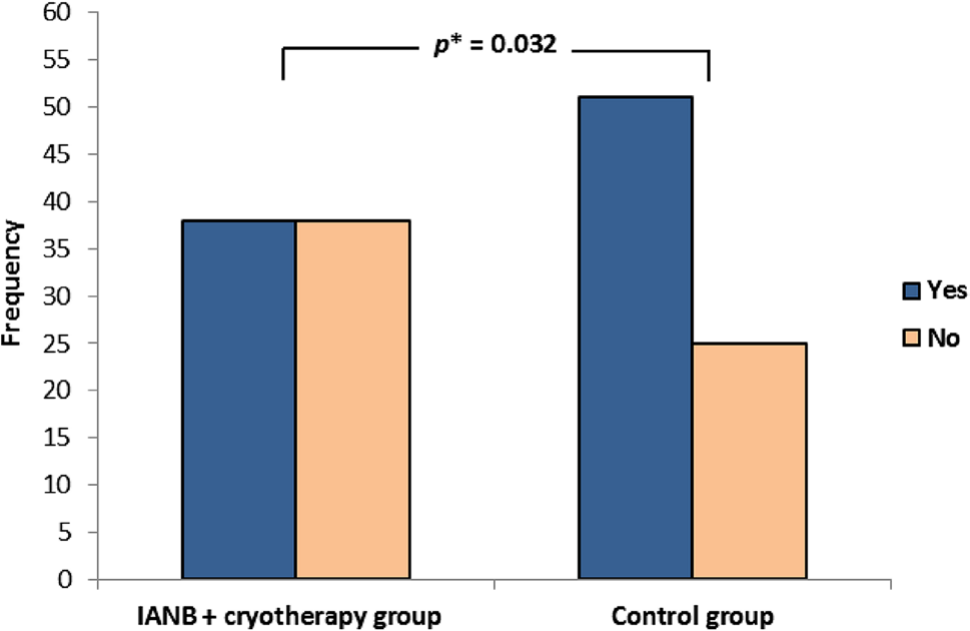
Frequency of the need of supplementary anesthesia (intraligemantary injection) in the two groups Inferior alveolar nerve block (IANB) *Chi-square test; *P*-value was set to 0.05

Figure 2 shows the need for additional ILI to obtain profound pulpal anesthesia. The frequency of extra ILI was 50% (*n* = 38) and 67.1% (*n* = 51) in the cryotherapy and control groups, respectively (*p* = 0.032).

## Discussion

Limited data are available regarding the IANB's efficacy to anesthetize the mandibular permanent teeth with SIP in patients younger than 18 years. The null hypothesis (*H0*) of the primary outcome assumed no difference in pain intensity during endodontic treatment of the mandibular first permanent molars with SIP after IANB injection with and without cryotherapy application. In terms of supplementary ILI, the null hypothesis assumed that there was no difference in the need for additional injection between the two groups. The findings of the current trial rejected the primary and secondary null hypotheses.

For the sake of standardization, only patients with closed mandibular first permanent molar apexes suffering from SIP were included in the trial. Ice packs for candidates in the experimental group were applied for 5 min unless a burning sensation or cold intolerance was reported. Our findings showed no adverse effects after cryotherapy application for 5 min. A similar application time was adopted in a previous study [10]. The application time was limited to a maximum of 5 min to avoid the risk of soft tissue damage [27]. More age-related research may be required to determine the effective timing of cryotherapy application without causing harm to the oral soft tissues. Meanwhile, Vera et al. recommended that 3–5 min of applied cryotherapy is efficient [14].

Several studies have stressed the positive association between the intensity of preoperative pain and intraoperative pain during endodontic treatment [28–32]. Therefore, an inclusive selection of participants complaining of preoperative moderate or severe pain levels was considered. Additionally, independent statistical analysis for patients with preoperative moderate and severe pain intensities was applied.

To control other factors that could influence the participant’s intraoperative pain, including operator factors, two pediatric dentists were responsible for IANB injection and ice pack placement independently, and a single experienced operator accredited to perform endodontic treatment and adopting identical preoperative restrictive inclusion standards were applied. To control patient- and teeth-related factors such as the preoperative pulp condition, the thermal and the EPT and patients without preoperative analgesic intake within the previous 12 h were exclusively included [33].

The average onset of pulpal anesthesia following IANB and 4% articaine with 1:100,000 epinephrine was varied. Some articles reported an average onset of 4.2 min [34], while others suggested an average of approximately 7 min [35, 36]. Another study reported a success rate of only 40% of subjects using the same concentration of articaine after 15 min of local anesthetic solution administration [37]. In the current study, a 20-min waiting interval was considered before starting the endodontic procedures to include all possible participants with delayed onset of pulpal anesthesia. Additionally, the presence of inflamed pulp tissues may delay the onset of pulpal anesthesia. This interval was adopted in a previous clinical trial that assessed the efficacy of an IANB of 4% articaine as a local anesthetic agent [6].

Before determining the volume of local anesthesia, two main considerations have been taken into account. First, patients with a minimum weight of 35 kg were included. This guaranteed not exceeding the maximum dose recommended by the American Academy of Pediatric Dentistry (AAPD) and avoiding systemic toxicity [38]. The other concern was the efficacy of 3.6 mL over 1.8 mL of the local anesthetic agent in the case of SIP, which has been proven. Several studies advocated the use of a higher volume of anesthetic solution in the pterygomandibular space to accelerate the filling of the space [10, 39, 40]. At the same time, the higher volume allows more exposure of the nerve to the anesthetic solution [40]. The findings of a previous study that included 80 patients suffering from SIP related to the mandibular first permanent molars showed that the success rate of 3.6 mL of articaine was 77.5% compared to 27.5% for 1.8 mL [41].

Pain intensity was measured using the VAS, which was accredited in the current study because of its adequate psychometric properties. The VAS is easily applied in clinical practice with excellent inter-observer and test–retest reliability, repeatability, acceptability, responsiveness, and validity [22, 42, 43]. Additionally, the VAS is sensitive to minor changes in pain intensity [22]. It is appropriate for children aged 10 and up [45] and has been widely used in previous studies [44–47].

Pain intensity in the cryotherapy group was significantly less than that in the control group. Our overall success rate in the cryotherapy group was 59.2%. This was in line with the overall success rate reported by Topçuoglu et al. [10]. They concluded that the use of cold applications improved the efficacy of IANB by up to 55.8% in adults’ teeth.

The mechanism of action of cryotherapy when applied to the mucosal soft tissues is to include vasoconstriction that lowers the cellular oxygen demand (i.e., hypoxia) and subsequently diminishes cellular metabolism, leading to less tissue damage [48]. Furthermore, cold minimizes tissue nociceptor stimulation and the speed of painful signal propagation [14]. These two actions are responsible for the short-term local analgesic effect of cryotherapy [48].

Our findings showed that the greater the extent of pulp inflammation, the lesser the efficacy of the IANB technique. The application of cryotherapy failed to enhance the efficacy of IANB when preoperative pain scores were severe. The difference between the two groups was insignificant. In the case of SIP, peripheral and central sensitization were exaggerated, resulting in higher pain thresholds than in normal pulp tissues [49].

The reported success rates of IANB in the mandibular teeth with SIP in patients below the age of 18 were varied. Chompu-inwai et al. [50] tested the effectiveness of IANB and ILI in 60 mandibular first permanent molars with deep caries. Out of 14 molars with irreversible pulpitis, only 3 teeth showed pulpal anesthetic success (i.e., a success rate of 21.4%), which was less than our success rate. This could be related to the smaller sample size and the difference in methods adopted to measure pain intensity, while another study conducted by Chompu-inwai et al. [51] reported a success rate of 48%. This was comparable to our success rate.

Regarding the secondary outcome results, the need for supplementary ILI was still mandatory in both groups. Meanwhile, it occurred significantly less frequently in the cryotherapy group than in the control group. The low success rate of routine IANB among teeth diagnosed with irreversible pulpitis increases the demand for supplementary anesthesia [52].

The main strengths of this study were the adequate sample size and the rigorous measures to ensure higher standardization qualities. The study’s limitation was the lack of subject masking of the nature of the treatment. Another problem could arise from the complex nature of pain. Some factors, including preoperative anxiety and different pain thresholds, may influence the subjective scoring system. However, the randomization process ensured equal distribution of different determinants. Finally, most of the available data, especially those related to cryotherapy, were related to adults’ teeth older than 18 years, therefore comparing our results to previously published data has to be taken into consideration. To cover this point, further prospective clinical trials need to be conducted with the same age group.

## Conclusions

Within the limitations of the current study, the following conclusions can be made:

1. Cryotherapy application boosted the efficacy of IANB in anesthetizing the mandibular first permanent molars with SIP and significantly diminished the intraoperative pain scores during endodontic treatment of the first permanent molars in adolescents.
2. The success rate of profound pulpal anesthesia is significantly higher in the cryotherapy group.
3. Additional anesthesia was still necessary for optimal control over pain. However, the frequency of the need for ILI in the cryotherapy group was significantly lower than that in the control group.

## Supplementary Information

Below is the link to the electronic supplementary material.

Supplementary file1 (DOCX 32 KB)

## Data Availability

The datasets used during the current study available from the corresponding author on reasonable request. All data analyzed during this study are included in this published article in the form of tables and figures.
