# Supplementary material for: Cryotherapy and pain intensity during endodontic treatment of mandibular first permanent molars with symptomatic irreversible pulpitis: A randomized controlled trial
Source: Clin Oral Investig. 2023 Jun 3;27(8):4585–93. doi: 10.1007/s00784-023-05084-1 (PMC10415469; doi:10.1007/s00784-023-05084-1)
Supplement: Supplementary file 1 — Supplementary file1 (DOCX 32 KB) [file 784_2023_5084_MOESM1_ESM.docx]

**PRIRATE 2020**

**Checklist of items to be included when reporting Randomized Trials in Endodontics***

.

| \| Section/ Topic \| Item number \| Checklist Items \| Reported on page number \| \| --- \| --- \| --- \| --- \| \| **Title** \| 1a \| The phrase “Randomized clinical trial” or “randomized controlled trial” must be included in the title. \| 1 \| \| 1b \| Details of the specific area(s) of interest using words and phrases that identify the clinical problem and the intervention(s), must be provided \| 1 \| \| **Keywords** \| 2a \| Keywords indicating the specific area(s) of interest using MeSH terms must be included \| 1 \| \| **Abstract** \| 3a \| The Introduction of the Abstract must explain briefly the rationale for the trial \| 1 \| \| 3b \| The aim/objective(s) of the trial must be provided at the end of the Introduction section within the Abstract \| 1 \| \| 3c \| The Methodology section within the Abstract must provide essential information on the nature of the trial (e.g. superiority, non-inferiority, equivalence), its design (e.g. parallel, split-mouth, crossover), the inclusion/ exclusion criteria, randomization process, blinding process and statistical analysis \| 1 \| \| 3d \| The Results section within the Abstract must describe the number of participants that were randomized and analyzed, the size and direction (group favoured) of the difference(s) between the intervention(s) and control groups with statistical analysis (p values and 95% CI). Adverse events or side-effects (if any) must also be reported or if none occurred, that must be mentioned explicitly \| 1 \| \| 3e \| The Conclusion section within the Abstract must summarise the findings and emphasise the clinical implication(s) of the results \| 1 \| \| 3f \| The prospective registration (number and name of the registry) and source(s) of funding must be provided \| Provided in title page \| \| Introduction \| 4a \| The scientific background and rationale for the trial must be provided, including the gap(s) or inconsistencies in knowledge \| 2&3 \| \| 4b \| The specific aim/objective (s) of the trial must be provided and the main clinical research question formulated clearly, preferably using the PICO framework (Problem/Population, Intervention, Control and Outcome) \| 3 \| \| **Methods**  *Trial Design* \| 5a \| Details of the nature of the trial (superiority, non-inferiority, equivalence of experimental intervention(s)), its design (parallel, split mouth, crossover, single/double blinded) and test:control allocation ratio must be provided. If applicable, important information about the study design must also be provided, e.g. pragmatic or preference trial, phase (drug trials), patient or public involvement in planning etc \| 4 \| \| 5b \| Changes to the methodology after the trial commenced (such as eligibility criteria) must be provided along with detailed explanations \| NA \| \| *a priori protocol* \| 5c \| Details of the ethical approval of the protocol and the process for obtaining informed consent must be provided \| 3&4 \| \| 5d \| Details of the trial protocol including registration number and name of registry/clinical database and where it can be accessed (open access webpage, if applicable) must be provided \| 3 \| \| *Participants (patients, operators, evaluators)* \| 5e \| A list of inclusion and exclusion criteria at the individual/tooth/root level must be provided \| 5&6 \| \| 5f \| Details of the setting/environment of the trial must be provided. Details on how many operators were involved in performing the intervention and control and their relevant experience/qualifications are essential. The setting where the data were collected must be described. If several operators are included and/or if it is a multi-centre set-up, details of how standardization/calibration between individuals or centres was achieved must be provided \| 6 \| \| 5g \| The treatments in the intervention (experimental) group(s) must be described with sufficient detail to allow replication, including how and when they were actually administered \| 7 \| \| 5h \| The intervention(s) or absence of intervention(s) in the control group must be described with sufficient details to allow replication, including how and when the intervention(s) was actually administered \| 7 \| \| *Outcomes measures* \| 5i \| The primary and secondary (if any) outcome measures must be described, including how and when they were assessed and by whom \| 8 \| \| 5j \| Details of any changes made to the study outcomes after the commencement of the trial must be described \| NA \| \| 5k \| If primary or secondary outcomes are to be regarded as surrogate outcomes, the rationale and empirical support for the connection between surrogate(s) and the outcome(s) of clinical relevance must be provided \|  \| \| *Sample size* \| 5l \| How the sample size was determined must be described with reference to the published literature, or a pilot study. The sample size may be modified after an internal feasibility study**.** Sample size calculations should generally refer to the primary outcome measure. If secondary outcome measures constitute the base for sample size calculation, an explanation must be provided \| 4 \| \| 5m \| Any interim analyses and stopping guidelines must be described, when applicable \| NA \| \| *Randomization and allocation concealment* \| 5n \| The method used to generate the random allocation sequence along with any details of the type of restriction (e.g. blocking) if applicable must be described. The persons responsible for randomization and recruitment must be provided. For multi-centre trials a central randomization procedure is preferred and must be described. The unit of randomization should be specified and justified. Any stratification variables must be detailed \| 4 \| \| 5o \| Methods for allocation concealment up to the assignment of the participants into the intervention groups must be described \| 4 \| \| *Blinding* \| 5p \| Information on who was/were blinded after assignment to the interventions (e.g. participants, care-givers, evaluators) must be described in detail. Blinding through masking of interventions (e.g. similar looking drugs/instruments) should be described. Detailed reasons for lack of blinding (if applicable) must be described \| 4 \| \| *Statistical analysis* \| 5q \| The statistical methods used for analysis of the primary and secondary (if any) outcomes, additional subgroup analyses and adjusted analyses (if applicable) must be described in detail. Consideration of drop-outs should be included in the calculations \| 9 \| \| 5r \| How any cluster effects were managed during the analysis must be described \| NA \| \| **9Results** \| 6a \| The number of participants who were randomly assigned, received the intended treatment, and were analysed for the primary and secondary (if any) outcome(s) for each group must be described. A flowchart must be provided \| 9 \| \| 6b \| Reasons for losses/drop-outs and exclusions after randomization must be described for each group and included in the flow chart. If intention to treat analyses are used, details of the process must be provided \| 9 \| \| 6c \| The dates of recruitment, follow-up and study duration must be described \| 9 \| \| 6d \| Reason(s) for any early termination of the trial must be described \| 9 \| \| 6e \| The baseline demographic and clinical characteristics of each group must be provided \| 9 and table 1 \| \| 6f \| The results for each group for each primary and secondary (if any) outcome(s), along with the estimated effect size and its precision, must be provided \| 9&10 \| \| 6g \| Both absolute and relative effect sizes for binary outcomes must be provided \| NA \| \| 6h \| The results from any other analyses performed must be described, including subgroup analyses and adjusted analyses, distinguishing pre-specified from exploratory \| 9 \| \| 6i \| The incidence and management of any adverse effects or unintended effects in each group must be described \| NA \| \| **Discussion** \| 7a \| An estimate of the overall internal validity must be provided as well as the generalizability (external validity, applicability, real-world relevance) of the trial findings \| 10-14 \| \| 7b \| The rationale for inclusion, exclusion criteria and study duration must be provided \| 10,11,12,13 \| \| 7c \| An explanation of the clinical relevance of the primary and secondary outcomes must be provided \| 10,13,14 \| \| 7d \| A detailed interpretation consistent with results, balancing benefits and harms, and considering other relevant evidence must be provided \| 10-14 \| \| 7e \| The strength(s) of the trial must be provided \| 14 \| \| 7f \| The limitations of the study must be provided, addressing the sources of potential bias, imprecision and, if applicable, multiplicity of analyses \| 14 \| \| 7g \| Implication for future research and clinical practice must be described \| 14 \| \| **Conclusion** \| 8a \| A rationale for the conclusion(s) must be provided, and the clinical significance highlighted \| 14 \| \| 8b \| Explicit conclusion(s) from the trial must be provided \| 14 \| \| **Funding details** \| 9a \| Sources of funding and other support (such as supply of drugs, equipment) as well as the role of funders must be acknowledged and described \| Figure 1 \| \| **Conflict of interest** \| 10a \| An explicit statement on conflicts of interest must be provided \| Provided \| \| **Quality of images (if applicable)** \| 11a \| Details of the equipment, software and settings used to acquire the image(s) must be described in the text or legend \| 9 \| \| 11b \| The reason why the image(s) was acquired and the rationale for its inclusion in the manuscript must be provided in the text. A justification for all images which involve radiation must be included \| 9 \| \| 11c \| The circumstances (conditions) under which the image(s) were viewed and evaluated by the authors must be provided in the text \| 9 \| \| 11d \| The resolution and any magnification of the image(s) or any modifications/enhancements (e.g. adjustments for brightness, colour balance, or magnification, image smoothing, staining etc) that were carried out must be described in the text or legend \| NA \| \| 11e \| Patient(s) identifiers (names, patient numbers) must be removed to ensure they are anonymised \| NA \| \| 11f \| An interpretation of the findings (meaning and implications) from the image (s) must be provided in the text \| 9&10 \| \| 11g \| The legend associated with each image must describe clearly what the subject is and what specific feature(s) it illustrates. Images of patients must describe the age, gender and ethnicity of the person, if relevant \| Applied \| \| 11h \| Markers/labels must be used to identify the key information in the image(s) and defined in the legend \| Applied \| \| 11i \| The legend of each image must include an explanation whether it is pre-treatment, intra-treatment or post-treatment and, if relevant, how images were standardised over time \| Applied \| |
| --- | --- | --- | --- | --- | --- | --- | --- | --- | --- | --- | --- | --- | --- | --- | --- | --- | --- | --- | --- | --- | --- | --- | --- | --- | --- | --- | --- | --- | --- | --- | --- | --- | --- | --- | --- | --- | --- | --- | --- | --- | --- | --- | --- | --- | --- | --- | --- | --- | --- | --- | --- | --- | --- | --- | --- | --- | --- | --- | --- | --- | --- | --- | --- | --- | --- | --- | --- | --- | --- | --- | --- | --- | --- | --- | --- | --- | --- | --- | --- | --- | --- | --- | --- | --- | --- | --- | --- | --- | --- | --- | --- | --- | --- | --- | --- | --- | --- | --- | --- | --- | --- | --- | --- | --- | --- | --- | --- | --- | --- | --- | --- | --- | --- | --- | --- | --- | --- | --- | --- | --- | --- | --- | --- | --- | --- | --- | --- | --- | --- | --- | --- | --- | --- | --- | --- | --- | --- | --- | --- | --- | --- | --- | --- | --- | --- | --- | --- | --- | --- | --- | --- | --- | --- | --- | --- | --- | --- | --- | --- | --- | --- | --- | --- | --- | --- | --- | --- | --- | --- | --- | --- | --- | --- | --- | --- | --- | --- | --- | --- | --- | --- | --- | --- | --- | --- | --- | --- | --- | --- | --- | --- | --- | --- | --- | --- | --- |

***From: Nagendrababu V, Duncan HF, Bjørndal L, Kvist T, Priya E, Jayaraman J, Pulikkotil SJ, Pigg M, Rechenberg DK, Vaeth M, Dummer P. (2020) PRIRATE 2020 guidelines for reporting randomized trials in Endodontics: a consensus-based development. *International Endodontic Journal* Mar 20. doi: 10.1111/iej.13294.**

**For further details visit: http://pride-endodonticguidelines.org/prirate/**
